# A case of secondary acute myeloid leukemia on a background of glycogen storage disease with chronic neutropenia treated with granulocyte colony stimulating factor

**DOI:** 10.1002/jmd2.12069

**Published:** 2019-07-23

**Authors:** Dina Khalaf, Heather Bell, David Dale, Vikas Gupta, Hanna Faghfoury, Chantal F. Morel, Anne Tierens, David A. Weinstein, Jiong Yan, Santhosh Thyagu, Dawn Maze

**Affiliations:** ^1^ Department of Medical Oncology and Hematology, Princess Margaret Hospital Cancer Centre University Health Network Toronto Ontario Canada; ^2^ Fred A. Litwin Family Centre in Genetic Medicine University Health Network and Mount Sinai Hospital Toronto Ontario Canada; ^3^ Department of Medicine University of Washington Seattle Washington; ^4^ Department of Pathology, Toronto General Hospital University Health Network Toronto Ontario Canada; ^5^ Glycogen Storage Disease Program University of Connecticut and Connecticut Children's Medical Center Hartford Connecticut

**Keywords:** acute myeloid leukemia, congenital neutropenia, cornstarch, G‐CSF, glycogen storage disease, granulocytic sarcoma

## Abstract

Congenital neutropenias due to mutations in ELANE, SBDS or HAX1 or in the setting of glycogen storage disease (GSD) which is caused by *SLC37A4* mutation, often require prolonged granulocyte colony stimulating factor (G‐CSF) therapy to prevent recurrent infections and hospital admission. There has been emerging evidence that prolonged exposure to G‐CSF in cases with congenital neutropenia other than GSD is associated with transformation to myelodysplastic syndrome/acute myeloid leukemia.

## 1 INTRODUCTION

Glycogen storage disease (GSD) is an autosomal recessive disorder caused by mutations in the *SLC37A4* gene on chromosome 11q23. Deficiency in the translocase leads to intracellular accumulation of glycogen and disturbed glycogenolysis and gluconeogenesis.^1^ Affected individuals require particular dietary regimens, which include uncooked cornstarch (UCCS) as a slowly metabolized, low glycemic source of glucose, which results in less insulin secretion and a lower risk of hypoglycaemia and lactic acidosis.^2^

Clinical features of type Ib GSD include hypoglycemia, lactic acidosis, hyperuricemia, hyperlipidemia, hepatomegaly, inflammatory bowel disease, and growth retardation.^3^ GSD type Ib is also associated with neutropenia and neutrophil dysfunction resulting in recurrent infections.^4^ The introduction of recombinant granulocyte colony‐stimulating factor (G‐CSF) has improved quality of life and survival to adulthood,^5^ however, an association with myelodysplastic syndrome (MDS) or acute myeloid leukemia (AML) has been observed in some cases with congenital neutropenia on G‐CSF^6^ (Table 1).

**Table 1:** Case reports

Reference number	Relevance to our case
11	The first reported case that has described the association between GSD, and AML, however, prior to G‐SCF era
12	The first reported case of a young woman with GSD who was treated with G‐CSF therapy who developed AML
14	Here the young patient with GSD who was on G‐CSF therapy had AML with abnormal karyotype. In this case they described monosomy 7 as an associated abnormal karyotype
13	This was the third reported case that highlighted the link between G‐CSF therapy in patients with GSD and AML
15	The authors of this article have linked telomere shortening with AML in patients with GSD receiving G‐CSF in face of the associated neutropenia

We present our experience in the management of a young female with AML on a background of type Ib GSD and chronic neutropenia treated with G‐CSF therapy for almost 25 years. We highlight the plausible association between G‐CSF and leukemia risk, in a condition that may inherently be associated with an increased risk of AML, and to describe the supportive care required during induction chemotherapy for a patient with this rare metabolic condition.

## 2 CASE REPORT

**Figure 1:**
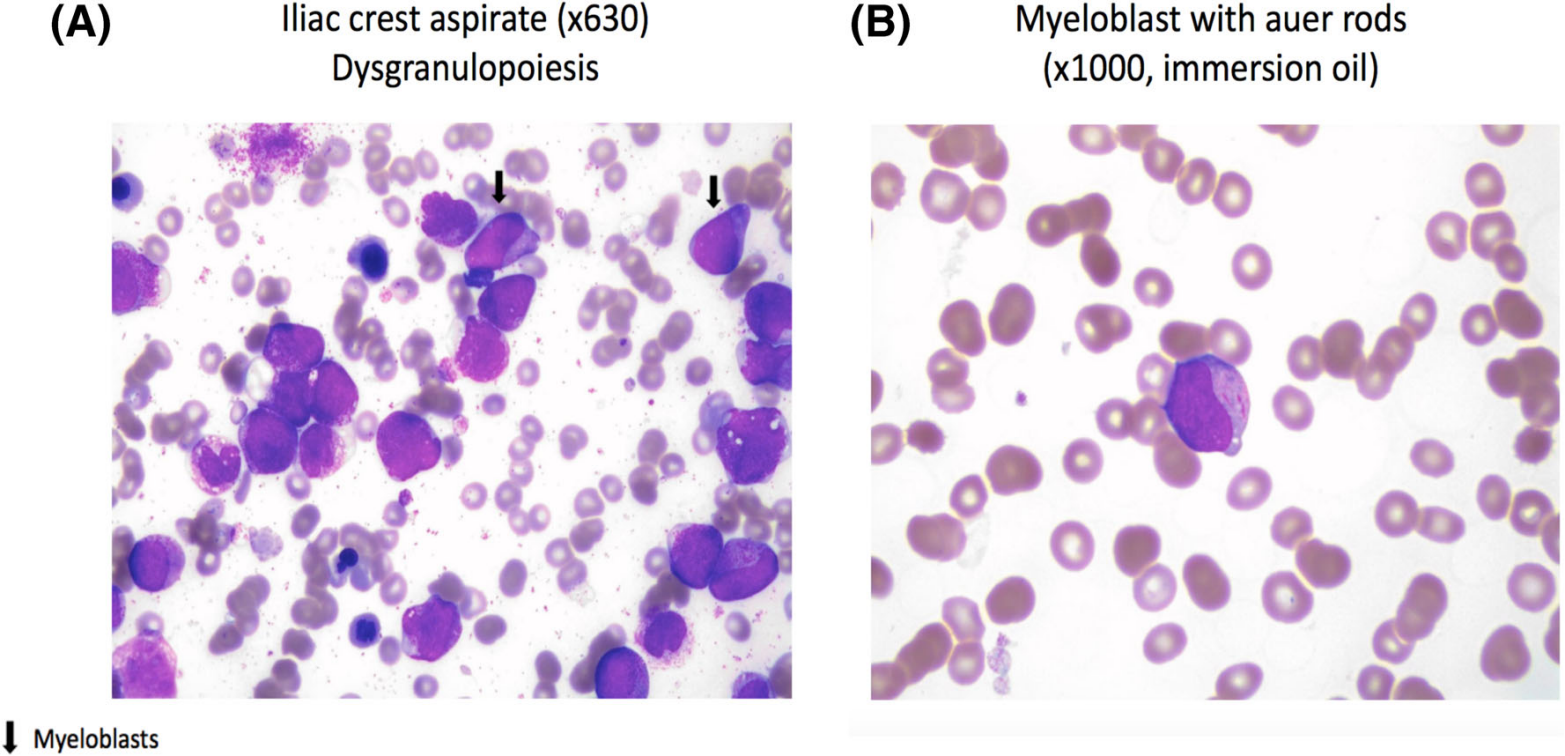
Bone marrow aspiration showing on the left (A) myeloblasts and dysgranulopoiesis; and on the right (B) showing a myeloblast with auer rod

Our patient was diagnosed with GSD as an infant. Weight‐based G‐CSF therapy was started at the age of 3 and reached 300 mcg daily by adulthood. Our patient was on a special diet that included oral UCCS every 3 to 4 hours during daytime and a carbohydrate solution administered at night time via a nasogastric tube. By age 26, the platelet count showed a mild decline in the range of 80 to 120 × 10^9^/L, which was felt to be from hypersplenism. Two years later, she developed recurrent oral ulcers, skin infections, weight loss, and low‐grade fevers. A CBC at that time showed Hb 112 g/L, WBC 2.4 × 10^9^/L, neutrophils 1.40 × 10^9^/L, and platelets 107 × 10^9^/L. Three months later, she developed pancytopenia with circulating blasts. A subsequent bone marrow aspiration demonstrated dysmegakaryopiesis, dyserythropiesis and 5% blasts with auer rods (Figure 1A,B).

Flow cytometry confirmed 5% to 6% blasts positive for CD117, CD13, CD33, CD38, and CD71, partially positive for CD7, CD34, CD64, CD65, and HLA‐DR, negative for CD11b, CD14, CD36, CD64, and CD123. There was decreased erythroid CD36 expression and aberrant maturation on CD11b vs CD16 scatter that confirmed a diagnosis of MDS‐EB2. A karyotype was normal and NGS showed NPM1 and NRAS mutations. This diagnosis was confirmed with a repeat bone marrow aspirate after G‐CSF had been interrupted for 2 weeks.

A low‐dose computerized tomography scan (CT) of the thorax to investigate unilateral pleuritic chest pain showed a pulmonary macro‐nodule with ground glass opacity and cavitation, suspicious for invasive aspergillosis infection. Fiberoptic bronchoscopy and bronchoalveolar lavage was planned, requiring the patient to fast overnight prior to the procedure. Considering her meticulous metabolic requirements, our team consulted the metabolic genetics team. Upon fasting, she was started on 10% dextrose in water with 0.45% saline (D10W‐0.45%NaCl) by intravenous infusion with serum glucose monitoring every 3 hours. The IV fluids were titrated to maintain glucose level between 4 and 6 mmol/L (70‐110 mg/dL). UCCS was reintroduced 2 hours following the procedure, and she was weaned off IV fluids without incident. Results of the BAL came back negative. Due to subsequent progressive worsening of the pulmonary cavity, a CT‐guided biopsy was performed and the pathology report revealed infiltration with myeloblasts: MPO+, CD117+ (30%), CD68+ less than 20%, CD34−, with no fungal elements. The bone marrow, however, did not show evidence of progression. She was diagnosed with a granulocytic sarcoma and the decision was made to proceed with induction chemotherapy using daunorubicin and cytarabine (3 + 7) prior to allogeneic SCT.

To address the unique metabolic considerations, the metabolic genetics team provided us with a protocol (Table 2). IV medications were preferentially dissolved in normal saline to reduce glucose accumulation. There were strict instructions to avoid administration of oral glucose or fructose to prevent sudden hyperglycemia and insulin secretion. Glucose monitoring was performed every 3 to 4 hours and nursing staff and trainees were educated on her metabolic requirements and to not administer: (a) glucagon due to risk of hypoglycemia and lactic acidosis, or (b) lactated Ringer's solution due to risk of lactic acidosis. A dedicated central venous access was inserted and reserved solely for the potential need for IV D10W in the event of fasting or symptoms that might hinder oral UCCS ingestion.

**Table 2:** Reference guide for dietary and metabolic management of GSD

THE PATIENT CANNOT FAST: PLEASE PLACE A SIGN OVER BED This patient has Glycogen Storage Disease, a condition that is caused by a defect in glucose‐6‐phosphate transporter, which prevents the liver from producing glucose from glycogen stores. There is a high risk of hypoglycemia and lactic/metabolic acidosis after a short term of fasting. The treatment of this condition is primarily dietary by supplying regular sources of glucose and AVOIDING FASTING. To facilitate this, please follow these guidelines. Every nurse, fellow, resident, medical student and all other team members directly involved in patient's care must read this document to maximize patient safety.
*Lab tests* On admission: CBC, glucose, electrolytes (sodium, potassium, bicarbonate, chloride), urea, creatinine, serum lactate, uric acid, AST, ALT, triglycerides, and cholesterol. Daily: glucose, electrolytes, AST, ALT, alkaline phosphatase, and lactate. Weekly: CBC, uric acid, cholesterol and triglycerides.
*Diet instructions* Usual diet order: No added sugar, low lactose, plus preferences. Ensure early breakfast tray ordered and all meals in room at scheduled times. 3 meals and 2 snacks with cornstarch daily, these must be taken every 2–3 h to avoid severe hypoglycaemia. Patient's family to be allowed to bring snacks from home as well as cornstarch supply. If the patient is unable to take nocturnal feeds or cornstarch, then run D10W with 0.45% NS at 110 mL/h overnight. Once the patient has eaten breakfast the next morning and tolerated 8 am cornstarch dose, taper infusion by 25 mL/h every 30 min until finished, then change to saline lock IV Note: Dose of cornstarch may need to be adjusted if the patient is febrile or undergoing active chemotherapy.
*Fasting instructions* ‐ Intravenous: If patient is NPO, initiate IV D10W with 0.45% NS and 20 mEq KCl at 110 mL/h. Check IV integrity every 2 h to ensure that IV fluid is still running. Once diet resumes and the patient has eaten a full meal with cornstarch, reduce IV rate to 50 mL/h for 1 h, then change to saline lock. If the patient requires TPN, please contact the TPN Genetic‐Metabolic Team
‐ Glucose monitoring: ‐ If the patient is eating, check blood glucose level prior to every cornstarch meal. ‐ If the patient is fasting or on IV fluids, check blood glucose level every 3‐4 h.
*Management of hypoglycemia* Glucose tablets: keep two packets at bedside to use p.r.n. as per table below: **Table:** Capillary blood glucose (mmol/L) *Action if patient eating* Less than 2.5 Give D50W 25 mL IV and recheck every 5 min until blood glucose is above 2.5 mmol/L, then follow instructions below 2.5 to 3.9 Give 1 glucose tab and recheck in 15 min; repeat if not above 4 mmol/L. When blood glucose level is above 4 mmol/L, give a carbohydrate snack. 4 to 12 No action required Greater than 12 NO insulin to be given; inform MD who will assess the route of intake (IV, PO, NG) *Action if patient is NPO and IV D10W is running* Give D50W 25 mL IV and recheck every 5 min until blood glucose above 2.5 mmol/L then follow instructions below
Important notes for team, pharmacy and nursing Try to minimize steroid use as this will cause significant rise in lactate. When necessary, use in lowest dose possible and as short duration as feasible, monitoring blood glucose, lactate and liver function q 4‐6 h rather than daily. Ensure medications are mixed in NS rather than D5 whenever possible to limit extra source of dextrose. Do not administer IV ringer's lactate at any time. Glucagon is to be avoided at all times (does NOT treat hypoglycaemia in GSD). Do not start IV D10W unless the patient is fasting, unable to eat due chemotherapy adverse effects. NEVER GIVE GLUCAGON TO TREAT HYPOGLYCEMIA IN PATINETS WITH GSD

**Figure 2:**
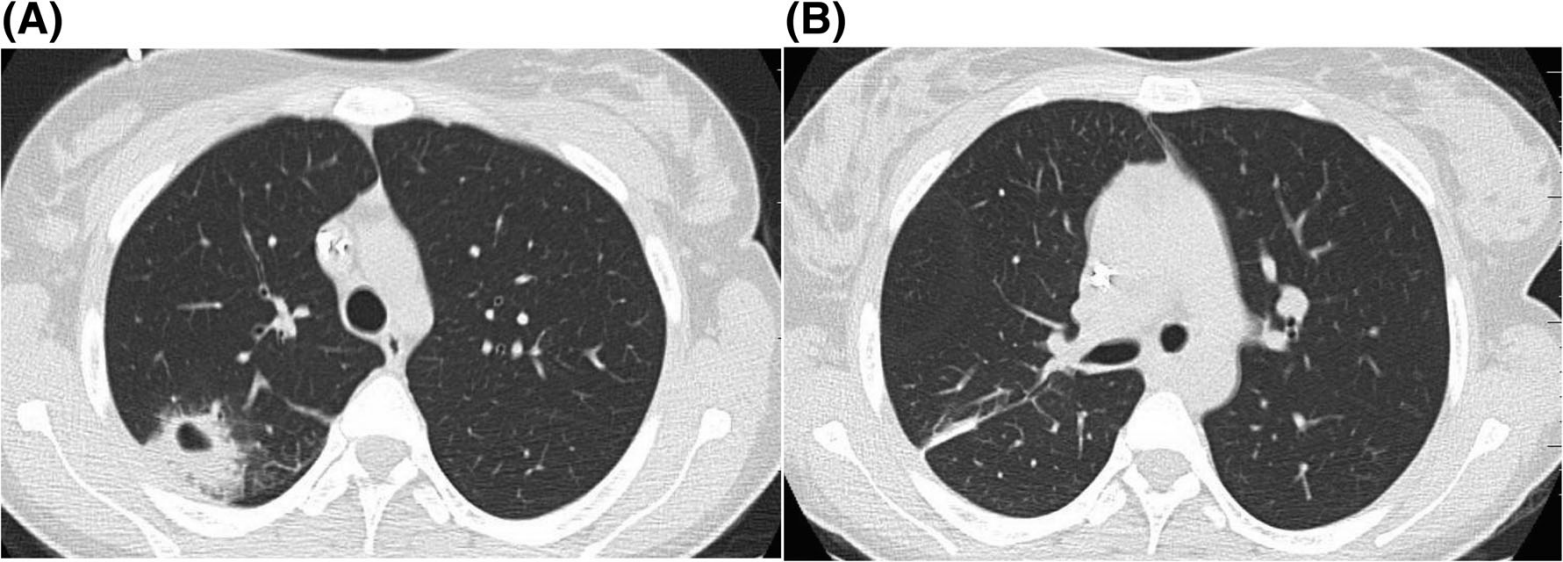
CT scans of the chest showing on the left. A, Preinduction myeloid sarcoma shown as a cavitary lesion with surrounding consolidation and ground glass; and on the right. B, A postoperative CT scan of the chest after wedge excision of the remnant lesion postinduction

Radiologic progression (Figure 2A) indicated a diagnostic surgical pulmonary wedge resection (Figure 2B). A similar strict metabolic protocol was followed due to fasting requirements. The excised tissue was free from residual disease. Bone marrow aspiration and biopsy showed a hypercellular marrow, granulocytic hyperplasia with left shift and some dysplasia with less than 4% blasts, indicating regenerative marrow. She proceeded to haploidentical SCT. To this date, the patient is doing well, almost 14 months posttransplant.

## 3 DISCUSSION

GSD type Ib is a rare congenital metabolic disorder that manifests with neutropenia and multiorgan dysfunction. The Severe Chronic Neutropenic International Registry (SCNIR) and The French National Severe Chronic Neutropenia Registry have both conducted individual prospective cohort studies where transformation to MDS/AML was documented in congenital neutropenia syndromes including GSD.^6^, ^7^, ^8^, ^9^ The SCNIR reported from their prospectively followed cohort 4 cases of AML in 83 patients followed for a mean of 12.2 years, that is, one case per 253 years of G‐CSF treatment, a lower rate than for patients with neutropenia due to mutations in *ELANE*, *SBDS* or *HAX1*.^6^, ^10^

A literature search identified four published case reports, with ours being the fifth (Table 1). Two were pediatric cases and the first preceded the era of G‐CSF therapy.^11^, ^12^ Three were adult cases, all exposed to G‐CSF (Table 1)^.^ 13, 14, 15 There is emerging evidence that prolonged use of G‐CSF in patients with GSD Ib may lead to marrow stress and telomere shortening^15^ which may promote for leukemogenesis. The risk may be increased by higher dose G‐CSF treatment, and low dose therapy has been recommended in this population.^1^, ^6^

While G‐CSF has been the standard therapy for the neutropenia in the GSD Ib population, there is increasing evidence that the bone marrow is not the primary problem. Several studies have demonstrated that bone marrow production of cells is normal, but apoptosis is occurring in the neutrophils resulting in premature death and neutropenia.^16^, ^17^ Vitamin E supplementation aimed at lowering oxygen radicals has been found to increase circulating neutrophil numbers and improve clinical outcome.^18^ More recently, Veiga‐da‐Cunha et al showed that failure to eliminate a phosphorylated nonclassical glucose analog (1,5AG6P) is causing inhibition of glycolysis, and treatment with a 1,5‐anhydroglucitol‐lowering drug successfully treated neutropenia in the murine model of GSD Ib.^19^

G‐CSF use in the GSD Ib population has been life sustaining, but it is now apparent that it is not treating the underlying disorder. Until new therapies are available, we recommend vigilant use of G‐CSF in this population. Patients with GSD appear to have a higher rate of complications from G‐CSF compared with other populations. Hypersplenism is almost universal even with low dosing, and the incidence of hematologic malignancies may be higher.^1^, ^6^ As a result of the tendency towards infective complications, low dose G‐CSF therapy has been recommended by consensus guidelines. Instead of 5 mcg/kg/day dose that is typically used in congenital neutropenia patients, doses administered range from 0.5 to 2 mcg/kg/day are typically used in the GSD Ib population.^1^ The consensus guidelines do not recommend bone marrow testing, but this recommendation may need to be reassessed in light of the growing number of cases of hematologic malignancies.

In addition to contributing to the growing literature on malignancies in GSD, this case report demonstrates the meticulous supportive care required for these patients. The stress of chemotherapy can induce a metabolic crisis, and strict attention to the metabolic requirements of patients with GSD is crucial to avoid hypoglycaemia and lactic acidosis.

## AUTHOR CONTRIBUTIONS

Dina Khalaf: first author, wrote up the manuscript, performed literature review and took the lead in circulating the draft among the co‐authors, obtained an informed consent form the patient, as well as submission for publication.

Heather Bell is a clinical dietician who collaborated with Drs. Faghfoury and Morel in drafting of the metabolic protocol (Table 2).

Drs. Vikas Gupta, David Dale, and David Weinstein reviewed and edited the manuscript. Drs. Tierens and Jiong from the hematopathology department provided the slides (Figure 1A,B), and critically reviewed the manuscript. Drs. Santhosh and Maze reviewed the final manuscript and helped with editing.

All of the above mentioned authors had substantial input in draft review, provided good follow up and critical edits to the manuscript, have approved the final draft and they agree to be accountable for this work.

## CONFLICT OF INTEREST

There are no competing interests to declare.

## PATIENT CONSENT

Patient consent has been obtained. There are no observations on their behalf.

## ETHICAL APPROVAL

Approval from the Institutional Committee for Care and Use of Laboratory Animals.
